# Targeted need’s assessment: Medical ethics in MBBS curriculum of Pakistan

**DOI:** 10.12669/pjms.35.5.873

**Published:** 2019

**Authors:** Arslaan Javaeed

**Affiliations:** Dr. Arslaan Javaeed, MBBS, M.Phil, MHPE. Department of Pathology, Poonch Medical College, Rawalakot, Azad Kashmir, Pakistan

**Keywords:** Medical ethics, Needs assessment, Undergraduate medical students

## Abstract

**Objective:**

To assess the learner need’s assessment of medical ethics in undergraduate medical curriculum of Pakistan.

**Methods:**

To establish an actual need, three methods were employed during October 2018. The first included a review of the curriculum for medical ethics as designed by the Pakistan Medical and Dental College (PMDC). A supplementary document “Code of Ethics”, published by Pakistan Medical and Dental College (PMDC), was also reviewed. In the second method, a self-administered questionnaire was distributed to all 500 undergraduate medical students at Poonch Medical College. Data analysis was performed through SPSS v 23.0 (IBM Corporation, Armonk, NY, US) at 95% CI. The results were expressed in the form of frequencies. The third method employed was an extensive review of literature to identify gaps and to propose learning strategies.

**Results:**

In the section on guiding principles in the curriculum, Ethics is considered as an optional subject. Bioethics is designated to be taught in the 3^rd^ year of the MBBS, as part of Forensic Medicine. The agreement to study Medical Ethics Principles as part of the curriculum among final-year medical students saw numbers almost double to 84.61%. The highest majority was seen among final year medical students where 84.6% of the students agreed to study principles of medical ethics as part of their curriculum.

**Conclusions:**

Data and the PMDC curriculum support the incorporation of medical ethics in undergraduate education. Thus, an effective educational program based on the assessment of needs could be developed for medical ethics.

## INTRODUCTION

In many medical colleges in Pakistan, medical ethics is not a part of their undergraduate curriculum. Currently, even though there is a prescribed syllabus for medical ethics, students are largely unaware of ethical issues such as confidentiality, non-maleficence, principles of autonomy, etc. As the students are deficient in their knowledge, this is reflected in their practice and later on, in their career as professionals. With the advancement of technology in medical health professions, widespread use of social media and multiculturalism, ethical issues in the medical profession are becoming more apparent. To cater for these challenges, medical students need to be taught extensively about medical ethics and how to deal with ensuing challenges.

According to Farquharson, a need implies a discrepancy or gap between a desired condition or state of affairs and the actual or perceived condition or state of affairs.^1^ Thus, it was a perceived need which was required to be established through formal means.^2^ As clinical rotations start in the 3^rd^ year of medical school with active interaction with patients, this needs’ assessment was targeted at 3^rd^ year medical students. Medical ethics is covered under the subjects of Forensic Medicine and Community Medicine, which are taught in 3^rd^ and 4^th^ years of the MBBS, respectively, therefore, the heads of both departments were involved, and the idea of a targeted needs’ assessment was discussed. Thus, the purpose of this student need’s assessment was to establish the need for knowledge of medical ethics among medical students.

## METHODS

To establish an actual need, three methods were employed during the month of October 2018. The first included a review of curriculum for medical ethics as designed by the Pakistan Medical and Dental College (PMDC). A supplementary document “Code of Ethics”, published by PMDC^3^, was also reviewed to enhance the understanding of the requirements of the medical council for newly graduated doctors. The M.B.B.S (Bachelors in Medicine, Bachelors in Surgery) curriculum, a 120-page document^4^, is to be followed by all the medical colleges in the country and the Code of Ethics is to be read, understood and agreed to be abided by all the medical graduates before registration with the Medical Council as practicing doctors. These documents would guide us to establish a local standard that the students have to achieve and will also help in defining educational programs according to it. Familiarity with the intended learning outcomes is defined as the first vital step in assessing learning needs.^5^

Riley states that students need to determine if there is an actual or perceived need rather than just a perceived need to learn a Code of Ethics so that the teaching process becomes more successful.^6^ To gather a perspective from the students about the need of medical ethics, a survey was conducted. A self-administered-questionnaire was distributed to all 500 undergraduate medical students studying in Poonch Medical College, Rawalakot, Azad Kashmir, Pakistan and collected once completed. Informed consent was secured from each participant and anonymity was ensured.

Ethical approval was secured from the institutional ethical board. Data analysis was performed through SPSS v 23.0 (IBM Corporation, Armonk, NY, US) at 95% CI. The results were expressed in the form of frequencies.

The third method used was an extensive review of literature, with the heads of both departments, to identify the information gaps and to propose learning strategies. This method not only provides an idea about the current situation of undergraduate medical education in terms of medical ethics but also suggests teaching strategies.^7^

## RESULTS

In the section on guiding principles in the curriculum, Ethics as an optional subject could be incorporated by the medical colleges. Bioethics as a subject is designated to be taught in 3^rd^ year of MBBS, as part of Forensic Medicine. Looking deeper into the curriculum of Forensic Medicine, it states that students should be able to understand and act according to the highest ethical standards in maintaining the doctor-patient relationship, patient examinations, and obtaining consent; and they should be knowledgeable about the laws pertaining to these matters. Medical ethics is also to be taught as part of subject of Community Medicine in the 4^th^ year where basic components, code of medical ethics and national recommended guidelines are stated as learning objectives. Ethics, including the rights of patients, was also included as part of psychiatry, which is taught in the final year. As part of the general learning objectives of all the clinical subjects such as medicine, surgery, ophthalmology etc., the students are expected to understand the principles of ethics pertaining to their particular specialty, and to maintain confidentiality. The review of this document shows that medical ethics is given importance in the curriculum and it is in 3^rd^ and 4^th^ year that students should learn about the principles of medical ethics and dealing with ethical issues and be able to apply this knowledge in their clinical placements.

The Code of Ethics specifically describes the ethical and moral duties of a physician practicing under the Pakistan Medical and Dental Council. Part 3 of the document, titled “Teaching ethics to students”^3^, states that sufficient measures should be taken by medical and dental colleges to ensure that medical ethics is being taught to the students with the goal of helping students identify, analyze and attempt to resolve common ethical issues. The information on this Code of Ethics, along with relevant journal articles, case studies, books, etc., should be made available to undergraduate students with specific emphasis on developing strategies to propagate information about ethics and ethical issues. In summary, documents emphasize that a good practical knowledge of medical ethics is expected of students as they enter into the professional field of medicine.

The next step was to define the need for Medical Ethics to be included in the curriculum perceived by students through conducting a survey. Out of 500 undergraduate medical students studying in Poonch Medical College, 473 agreed to complete the questionnaire, and 441 returned the completely filled questionnaires, making the response rate 88.2%. Informed consent was secured from each participant. Only 98 (22.22) were aware of the core principles of medical ethics (Table-I). Two hundred ninety-two (66.21%) students agreed that medical ethics should be taught in the undergraduate medical curriculum. The agreement to study medical ethics principles as part of the curriculum almost doubled from 49.46% among first-year medical students to 84.61% among final-year students, as shown in Table-II. When asked whether medical ethics should be taught through workshops instead of integration in the medical curriculum, only 89 students (20.18%) responded “yes”, as shown in Table-III. One hundred and thirty-three students (30.15%) preferred to study this subject in their final year while 45 students (10.15%) preferred to study medical ethics in first year (Table-IV). When given the options of teaching strategies of Problem Based Learning (PBL), Tradition Lectures and Self Study, 267 (60.54%) of students preferred PBL, as depicted in Table-V.

**Table I:** Response of the medical students regarding knowing the principles of medical ethics.

Do you know the principles of Medical Ethics?		

Year	Yes n (%)	No n (%)
First Year (n=93)	05 (5.37)	88 (94.62)
Second Year (n=81)	07 (8.64)	74 (91.35)
Third Year (n=87)	14 (16.09)	73 (83.90)
Fourth Year (n=89)	28 (31.46)	61 (68.54)
Final Year (n=91)	44 (48.35)	47 (51.64)

Total	98 (22.22)	343(77.77)

**Table II:** Response of participants regarding medical ethics course to be the part of their curriculum.

Do you think Medical Ethics should be part of the under-graduate medical curriculum?			

Course	Agreed n (%)	Disagreed n (%)	Don’t Know n (%)
First Year (n=93)	46 (49.46)	40 (43.01)	07 (7.52)
Second Year (n=81)	51 (62.96)	24 (29.62)	06 (7.40)
Third Year (n=87)	56 (64.37)	19 (21.83)	12 (13.79)
Fourth Year(n=89)	62 (69.66)	26 (29.21)	01 (1.1)
Final Year (n=91)	77(84.61)	14 (15.38)	00 (0)

Total	292 (66.21)	123 (27.89)	26 (5.89)

**Table III:** Showing response of the students whether medical ethics should be taught through workshops instead of integration into curriculum.

Do you think Medical Ethics should be taught through workshops instead of integrating it in the undergraduate medical curriculum?		

Year	Yes n (%)	No n (%)
First Year (n=93)	23 (24.73)	70 (75.26)
Second Year (n=81)	14 (17.28)	67 (82.71)
Third Year (n=87)	29 (33.33)	58 (66.66)
Fourth Year (n=89)	18 (20.22)	71 (79.77)
Final Year (n=91)	05 (5.49)	86 (94.50)

Total	89 (20.18)	352(79.81)

**Table IV:** Showing preference of medical students regarding the year in which medical ethics should be taught.

In which year should Medical Ethics be taught?					

Year	1^st^ Year	2^nd^ Year	3^rd^ Year	4^th^ Year	Final Year
First Year(n=93)	23 (25.27)	16 (17.20)	12 (12.90)	15 (16.12)	27 (29.03)
Second Year (n=81)	09 (11.11)	11 (13.58)	07 (8.64)	24 (29.62)	30 (37.03)
Third Year (n=87)	05 (6.17)	09 (10.34)	32(36.78)	29 (33.33)	12 (13.79)
Fourth Year (n=89)	02 (2.24)	13 (14.60)	26(29.21)	35 (39.32)	13 (14.60)
Final Year (n=91)	06 (6.59)	17 (18.68)	05 (5.49)	12 (13.18)	51 (56.04)

Total	45 (10.20)	66 (14.96)	82 (18.59)	115(26.07)	133(30.15)

**Table V:** Showing students preferred teaching strategy for medical ethics course.

What should be your preferred teaching strategy for Medical Ethics?			

Year	Problem Based Learning	Traditional Lectures	Self-Study
First Year (n=93)	48 (51.61)	40 (43.01)	05 (5.37)
Second Year (n=81)	45 (55.55)	25 (30.86)	11 (13.58)
Third Year(n=87)	42 (48.27)	39 (44.83)	06 (6.89)
Fourth Year (n=89)	60 (67.41)	21 (23.59)	08 (8.99)
Final Year (n=91)	72 (79.12)	18 (19.78)	01(1.09)

Total	267 (60.54)	143 (32.42)	31 (7.02)

Lastly, a review of literature with expert analysis was performed to assess the current situation of medical ethics in undergraduate medical education. A study carried out in Pakistan revealed that although the PMDC directs all universities to incorporate medical ethics into their curriculum and carry out assessments, most universities fail to do so. The main reasons were a lack of a centralized curriculum on medical ethics, no specified teaching methodology, and a lack of a formal assessment of students dealing with ethical issues.^8^ Another study carried out a thorough analysis on undergraduate medical curricula about ethics and stressed the need for curricular changes by considering medical ethics as a separate subject with allocated time and suitable learning resources, teaching strategies centered on contextual content and practice, continued assessments and desired competencies stated in the curriculum, so that effective learning can occur.^9^ Another study that focused on practicing physicians emphasized the importance of formal teaching of ethical issues in the clinical workplace to gain competence and which could be incorporated in the undergraduate medical curriculum in clinical years.^10^ Another factor highlighted is the importance of relevant contextual education and reasoning in medical ethics so that students are fully prepared to resolve any local ethical issues when confronted with them.^11^

## DISCUSSION

Three hundred forty-three (77.77%) students did not know about the principles of medical ethics, which is a shocking statistic. Around 66.21% (292) of the students agreed that it should be part of the undergraduate medical curriculum whereas 123 students did not agree. This implies that there are students who are in the “blind” quadrant of the Johari window and there is a role for educators, through evaluation and feedback, to help students move into the “open” quadrant.^5^ One of the reasons more students chose their final year to study medical ethics is because, as they near their graduation, they want to be experts in reasoning with ethical issues and to be comfortable with them. This survey identified that the majority of the students share a common understanding about the need of medical ethics.

As there was a dearth of studies on medical ethics in Pakistan, studies from neighboring countries were also reviewed. Teaching of medical ethics to undergraduate students was also rated as highly important by the general physicians of Iran in a needs assessment study.^12^ Another study, conducted in India, identified similar issues in Indian undergraduate medical curricula where medical ethics has recently begun to be taught as a separate course in some universities.^13^ An international survey carried out to assess the ethical curricula in Asian medical schools revealed that 89% of the medical schools provided an medical ethics course in some form in their curriculum.^14^ A study utilizing focus group interviews, conducted in Turkey, indicted that students had a strong understanding about the necessity of learning medical ethics in pre-clinical years.^15^ The students also preferred case-based discussions and had contradictory views on assignments or didactic lectures, which corresponds to the results of our survey.

Medical ethics is part of core curriculum in undergraduate medical education in the U.K, USA, Canada and Australia.^16^ An audit of ethics learning was carried out at a medical school through a secure website over one academic year to determine the quantity and range of medical ethics learning in the undergraduate curriculum and compare this with topics for teaching described by the Institute of Medical Ethics (IME Although 73% of medical schools in the U.K. taught all Institute of Medical Ethics recommended elements as part of their undergraduate medical ethics curricula, it is not being followed in all of the schools.^17^ The objectives of the medical ethics education, proposed teaching strategies and assessment methods are described in detail, in medical schools across United States.^18^

National Bioethics Committee (NBC) has now finalized a curriculum for teaching medical ethics in undergraduate medical and dental colleges.^19^ It has also suggested how to teach and assess the students. The final document was submitted to PM&DC which did approve it but it is not yet implemented.

### Recommendations

Research asserts that becoming aware of a need is pointless until strategies are utilized to meet it.^20^ The proposed educational strategies, in collaboration with the Department of Forensic Medicine and the Department of Community Medicine, would include a dedicated course on Medical Ethics in the teaching schedule of the students’ 3^rd^ year, including the code of ethics, and laws governing ethical issues. The students should initially concentrate on learning and then be provided with ethical issues to discuss. Role play, simulation and reflection can be preferred teaching strategies. Clinical supervisors can be informed about the findings of the needs assessment so that students can benefit from learning in a clinical environment and can be assessed objectively. A formal assessment must be incorporated at the end of the session. An evaluation can be conducted to see if the educational strategies are effective towards alleviating the identified needs.

## CONCLUSION

Assessing students’ needs is an important step in the creation of an effective educational program. The needs for inclusion of medical ethics in Pakistan’s medical school curricula focused on undergraduates. Primary data included a review of the MBBS curriculum, code of ethics and a survey. Secondary data included a detailed review of literature. It has been established that both teachers and students acknowledge there is a need for medical ethics to be part of the curriculum. Extensive data and the PMDC curriculum support the incorporation of medical ethics in undergraduate education with key emphasis on contextual content, case-based teaching methodology and formal assessments for ongoing learning.

## References

[1] Farquharson A, Assessing what needs to be learned (1995). Teaching in practice: How professionals can work effectively with clients, patients, and colleagues.

[2] Javaeed A, General Needs Assessment of the Undergraduate Medical Students to Integrate Courses on Medical Ethics, Time Management and Communication Skills into the Bachelor of Medicine, Bachelor of Surgery Curriculum of Pakistani Medical Colleges (2019). Cureus.

[3] Code of Ethics (2002). Pakistan Medical and Dental Council.

[4] (2016). Curriculum of M.B.B.S. Pakistan Medical and Dental Council.

[5] McKimm J, Swanwick T (2009). Assessing learning needs. Br J Hosp Med.

[6] Riley T (2014). Assessing clinical staff learning needs, Part 1. Clin Nurse Spec.

[7] Pilcher J (2016). Learning Needs Assessment. J Nurses Prof Dev.

[8] Farhan A, Majeed N, Mustafa AR, Azeem M, Waqar S (2018). Awareness of medical ethics in undergraduate medical students - A literature review. Awareness of Medical Ethics. Pak Armed Forces Med J.

[9] Shaikh A, Humayun N Medical ethics in undergraduate medical education in Pakistan:Towards a curricular change. Contemporary Issues in Bioethics. Croatia.

[10] Jalal S, Imran M, Mashood A, Younis M (2018). Awareness about Knowledge, Attitude and Practice of Medical Ethics pertaining to Patient Care, among Male and Female Physicians Working in a Public Sector Hospital of Karachi, Pakistan - A Cross-Sectional Survey. Eur J Environ Public Heal.

[11] Shamim MS, Baig L, Torda A, Balasooriya C (2018). Culture and ethics in medical education:The Asian perspective. J Pak Med Assoc.

[12] Asghari F, Samadi A, Rashidian A (2013). Medical ethics course for undergraduate medical students:A needs assessment study. J Med Ethics Hist Med.

[13] Ramesh Kumar K (2009). Ethics in medical curriculum;Ethics by the teachers for students and society. Indian J Urol.

[14] Miyasaka M, Akabayashi A, Kai I, Ohi G (1999). An international survey of medical ethics curricula in Asia. J Med Ethics.

[15] Can Bilgin A, Timbil S, Huseyin Guvercin C, Ozan S, Semin S (2018). Preclinical Students`Views on Medical Ethics Education:A Focus Group Study in Turkey. Acta Bioeth.

[16] Johnston C, Mok J (2015). How medical students learn ethics:an online log of their learning experiences. J Med Ethics.

[17] Brooks L, Bell D (2017). Teaching, learning and assessment of medical ethics at the UK medical schools. J Med Ethics.

[18] Carrese JA, Malek J, Watson K, Lehmann LS, Green MJ, McCullough LB (2015). The Essential Role of Medical Ethics Education in Achieving Professionalism. Acad Med.

[19] http://nbcpakistan.org.pk/assets/may-16-bioethics-facilitator-book---may-16%2c-2017.pdf.

[20] Walsh K (2006). How to assess your learning needs. J R Soc Med [Internet].

